# A study of emotional intelligence in an Egyptian sample of offspring of patients with schizophrenia

**DOI:** 10.1186/s43045-022-00216-x

**Published:** 2022-06-28

**Authors:** Tarek M. K. ElSehrawy, Eman Abo Elela, Ghada A. M. Hassan, Marwa El Missiry, Sohayla Abdel Nabi, Mahmoud Farag Soliman

**Affiliations:** 1 Ain Shams University, 38 Abbasia Square, Cairo, 1181 Egypt; 2 Galala University, Attaka, Egypt

**Keywords:** Offspring, Schizophrenia, Emotional intelligence, Social cognition, TEIQue

## Abstract

**Background:**

Emotional intelligence is usually a construct measured in healthy children, now it may be used for relatives of schizophrenia, and considered as trait marker for schizophrenia. Offspring of parents with schizophrenia are considered children with high familial risk for major mental disorder. The aim of the study is to assess emotional intelligence in a group of off springs of a parent with schizophrenia and compare them to healthy control subjects, and to find possible relation between emotional intelligence in offspring and profile of symptoms in schizophrenic parents.

**Results:**

Offspring of parents with schizophrenia had lower scores of emotional intelligences than their matched controls in emotion perception, self-esteem, low impulsivity and emotion regulation’s subsets of TEIQue-CF. There was correlation between offspring trait emotional intelligence and their parent’s duration of illness. There was no correlation found between schizophrenia severity in the parents and their offspring’s trait emotional intelligence.

**Conclusions:**

Offspring of parents with schizophrenia had impaired trait emotional intelligence in some of its facets when compared to normal healthy subjects.

## Background

Schizophrenia should not be considered an unavoidably inevitable deteriorating illness with poor determinism toward disability [1]. There is no population based prevention for schizophrenia; however, selection of asymptomatic high-risk population is the only hope [2]. Research within last decade had focused on offspring of high risk with family history of mental illness. Functioning in high-risk children is highly determined by social cognitive abilities [3].

Research on social cognition in schizophrenia has centered on five systems: emotion processing, social perception, social knowledge, theory of mind, and attribution style [3]. The most extensively studied aspect of social cognition in schizophrenia is emotion processing, which refers broadly to perceiving, understanding, and managing emotion in oneself and others [3]. One construct developed to measure emotion processing is “emotional intelligence” developed firstly by Mayer and Salovey [4]. There are 2 models explaining EI: trait and ability model. The advantage of the trait model as it measures the self-efficacy emotional perceptions rather than emotions as cognitive abilities [5].

Offspring of a parent with schizophrenia was known to have difficulties in socio-emotional interactions that may persist into adulthood [6].

Trying to assess the social cognition in high-risk offspring is challenging, however, few studies tried to measure emotional intelligence in offspring of parents with schizophrenia. Few, but only, rare studies which examine correlation between trait emotional intelligence and schizophrenia symptoms. Of those, Khatuna and Taha (2016) studied whether the trait EI affected in patients with schizophrenia as a part of emotional disturbance occurring and whether it is related to certain symptom cluster. They found EI is negatively correlated to primary symptoms of schizophrenia [7].

It was hypothesized that offspring of parents with schizophrenia may have impaired trait EI relative to normal healthy control subjects. And also, trait emotional intelligence of the offspring may be negatively affected by the clinical picture of schizophrenia in parents including duration of illness, severity and profile of symptoms.

Therefore, the objective of this study was to (1) measure emotional intelligence in offspring of parent with schizophrenia and compare them to matched group of healthy control subjects. (2) To assess relationship between clinical picture of schizophrenia in the parent and their offspring trait emotional intelligence.

### Subjects and methods

#### Study design

Cross-sectional case control study, carried on 50 offspring of parent with schizophrenia matched with 50 healthy typically developing children. The study was conducted in Okasha Institute of Psychiatry Ain Shams University Hospitals, Cairo, Egypt. Cases were collected from inpatient and outpatient, adult, and child clinics.

#### Subjects

A sample size of 50 participants was calculated to achieve 80% power to detect an effect size of 0.3000 using a 2 degrees of freedom chi-square test with a significance level (alpha) of 0.050 (confidence level 95%).

Both genders, all offspring of a parent, 6–12 years old, physically healthy, IQ above 70, no apparent documented neurological disorder, after exclusion of autism spectrum disorder by clinical history and examination, were enrolled in the study. Developmental history excluding any developmental abnormalities was taken. Reliable caregiver, either parent with the illness or the other parent, gave written informed consent.

#### Controls

Typically developing children of parents with no mental illness were enrolled after exclusion of intellectual disability, current mental disorder at the time of enrollment. Children were recruited through online survey presented to their parents. Only those matched with offspring as regards age, sex, social class and educational level were involved.

Inquiry about developmental milestones was made and only children with adequate development in motor, language, and social milestones were recruited as controls.

#### Tools and procedures

1. *Clinical data*: data collection about offspring and controls regarding socio-demographic data, developmental history, family history.
2. *Clinical data from the parent*: age, sex, duration of illness, number of hospitalizations, age of the child at the illness onset.
3. Arabic version of Structured Clinical Interview for DSM-IV Axis I Disorders (SCID-I) for diagnosis of schizophrenia in the parent. It usually takes between 1 and 2 h depending on the complexity of the past and current symptoms [8, 9].
4. *Positive and Negative Syndrome Scale (PANSS*) clinical interview for the parent. For assessment of schizophrenia severity in the parent. It is a 45-min clinical interview. The patient is rated from 1 to 7 on 30 different symptoms based on the interview as well as reports of family members or primary care hospital workers [10].
5. Arabic form of trait emotional intelligence scale child form (TEIQue-CF) [3]. It has been specifically developed as self-rating scale for children aged between 8 and 12 years. The scale comprises 75 items responded to on a 5-point scale and measures nine distinct facets which are emotional expression, emotional perception, emotional regulation, adaptability, affective disposition, self-esteem, self-motivation, low impulsivity, and peer relations. It was shown that the TEI has satisfactory levels of internal consistency (*a* = 0.76 and *a* = 0.73, respectively) and temporal stability [*r* = 0.79 and *r* (corrected) = 1.00]. Internal consistency for Arabic version used in this study was *a* = 0.73. The collected data was rated by the developers of the scale.
6. T*he Wechsler Intelligence Scale for Children (WISC)* for the general intellectual ability for the children. For exclusion of intellectual disability below 70 as inclusion criteria.
7. *Statistical analysis:* the collected data was revised, coded, tabulated and introduced to a PC using Statistical package for Social Science (SPSS 25). As regards descriptive statistics: mean, standard deviation (± SD), and range for parametric numerical data, while median and interquartile range (IQR) for non-parametric numerical data and frequency and percentage of non-numerical data were used. Student’s *t* test was used to assess the statistical significance of the difference between two study group means. *Mann–Whitney test* *(U test)* was used to assess the statistical significance of the difference of a non-parametric variable between two study groups. *Chi-square test* was used to examine the relationship between two qualitative variables. *Fisher’s exact test* was used to examine the relationship between two qualitative variables when the expected count is less than 5 in more than 20% of cells. *P* value: level of significance, was judged as follows: *P* > 0.05: non-significant (NS). *P* < 0.05: significant (S). *P* < 0.01: highly significant (HS).

## Methods

Children of adults admitted with schizophrenia were recruited through their reliable caregiver contact, and adults with schizophrenia attending OPC were asked to set a date for their children assessment for the study. From the period between December 2020 and December 2021, offspring who met inclusion criteria were recruited. Initially, 70 participants were asked to join this study, 12 children were excluded due to being intellectually disabled their IQ below 70, the other 8 children were collected by telephone calls screening but unfortunately they were dropped out due to difficulty of attending hospital at the time period of the study. So, only 50 were included fulfilling inclusion criteria.

The work divided into two phases**: **phase 1 was interviewing the parent, asking for the diagnosis, taking clinical history, and collecting signs of schizophrenia present at the time of interview by SCID-I and PANSS. Phase 2 is setting a date arranging for the child assessment of IQ and TEIQue-CF.

## Results

### Descriptive data

#### Socio-demographic data of both groups

The mean age of offspring population in the study was 9.76 ± 1.93 years old with median 10 years (IQR 8–11). This was matched to the mean age of the control group which was 8. 87 ± 2.12 years old (*t* = 0.544 *p* value; 0.293).

The offspring group included 34 (68%) females while 16 (32%) males matching the control group which included 30 (60%) female while 20 (30%) males (*χ*^2^ = 0.694, *p* value 0.404). As regards sex distribution in the offspring group, female to male ratio of 1:1.5 (Table 1).

**Table 1:** Sociodemographic data of both study groups and correlation with their total trait emotional intelligence score

Parameter		Group				Test of significance		
		Offspring (*N* = 50)		Control (*N* = 50)		*T* test	*P* value	Significance
		Mean/*N*	SD	Mean	SD			
Sex	Males	16	32%	20	30%	*χ*^2^ = 0.694	0.404	NS
	Females	34	68%	30	60%			
Age		9.96	1.93	8. 87	2.12	*t* = 0.544	0.293	NS
Trait EI global score		2.97	0.52	2.9	0.09	− 0.988	0.328	NS

*SD* standard deviation, *N* number, *S* significant, *NS* not significant, *T* Student’s *t* test of significance (*t* = Student’s *t* test value). *C* chi-square test of significance (*χ*.^2^ = chi-square test value) EI emotional intelligence

Table 1 shows socio-demographic data of both study groups: the offspring of schizophrenia and their healthy-matched children, and comparing their total score on trait emotional intelligence test

#### Parent clinical characteristics

The mean age of parents with schizophrenia at the time of examination in cases population were 40.94 ± 8.05 with a median age of 40 (35–45), minimum age of 29 years old, and maximum age of 55 years old. Only the age of parent with schizophrenia was included whether mother or father.

While the mean age of the child at the onset of parental illness was found to be 2.52 ± 5.56, and the median was 0 years old which means the illness started since the child was born (IQR 0–3).

The mean duration of parent illness is 13.52 ± 6.86 years. The median duration of illness was 10 years (IQR 6–20).

Twenty (40%) cases had a parent who was hospitalized once during their lifetime, while 16 (32%) had a parent hospitalized twice. only 14 cases (28%) had parent who was never hospitalized.

Parental schizophrenia was assessed using PANSS score. The mean total PANSS score found in the parent’s group was 37.82 ± 15.08 and the median score was 40.5 (IQR 25–44).

As regards positive symptom domain of PANS, 35 (70%) parents had delusions, 22 (44%) has suspiciousness/persecution, and only 19 (38%) had hallucinatory behavior.

As regards negative symptoms, the most common negative symptom found was emotional withdrawal; the least common symptom found in the parents was difficulty in abstraction.

As regards general psychopathology subscale, 23 (46%) parents had motor retardation, 16 (32%) parents had anxiety, and 16 (32%) had disturbance of volition.

Nineteen (38%) parents were diagnosed with undifferentiated schizophrenia, 17 (34%) were diagnosed with residual schizophrenia, while 11 (22%) were found to be paranoid, and only 3 (6%) were disorganized.

##### Comparison between trait emotional intelligence (global score and subtypes) of offspring of parents with schizophrenia and healthy control subjects

As regards the median global score of trait emotional intelligence-child form (main variable of this study) was 2.93 ± 0.37 with range of (2.13–3.8).

Comparing both groups regarding TEIQue, there was highly significant difference between both groups regarding subtests as emotion perception, self-esteem, low impulsivity, and emotion regulation. However, there was no significance difference in total score of the scale (Table 2).

**Table 2:** Comparison between emotional intelligence of offspring of parents with schizophrenia and their matched healthy controls as regards global score of TEIQue

	Cases	Control	*t* test		
	Mean ± SD	Mean ± SD	***t***	*p* value	sig
Adaptability	3.04 ± 0.73	3.13 ± 0.38	0.719	0.475	NS
Emotion expression	2.84 ± 0.68	2.7 ± 0.34	− 1.239	0.219	NS
Emotion perception	2.63 ± 0.25	3.03 ± 0.65	− 4.071	< 0.001	S
Self-motivation	3.22 ± 0.44	3.23 ± 0.44	0.054	0.957	NS
Self esteem	2.88 ± 0.36	3.25 ± 0.69	− 3.383	0.001	S
Low impulsivity	2.92 ± 0.48	2.6 ± 0.97	2.1	0.039	S
Peer relations	3.03 ± 0.9	2.89 ± 0.19	− 1.099	0.277	NS
Emotion regulation	2.6 ± 0.28	2.86 ± 0.46	− 3.376	0.001	S
Affective disposition	2.9 ± 0.88	3.12 ± 0.21	1.686	0.097	NS
Total TEIQ score	2.97 ± 0.52	2.9 ± 0.09	− 0.988	0.328	NS

##### Relation between trait emotional intelligence in the offspring and the clinical characteristics of schizophrenia in the parent (age, onset, duration of illness, and number of hospitalizations)

It was found that the trait emotional intelligence global score of the offspring is affected by their parent’s duration of illness found to be significant (*R− *0.296, *P* value 0.037), while the offspring emotional intelligence is not affected by the severity of symptoms of schizophrenia in the parent, the number of hospitalizations and the age of the offspring at which the illness started (Table 3).

**Table 3:** Statistical correlation between trait emotional intelligence and clinical characteristics of the parent with schizophrenia

Parameter	Total TEIQ score	Test of significance		
		Pearson correlation	*P* value	Significance
Parental age		− 0.175	0.224	NS
Total PANSS score	Total positive score	0.089	0.538	NS
	Negative score	0.097	0.503	NS
	General psychopathology	− 0.145	0.316	NS
	Total PANSS	0.005	0.975	NS
Duration of illness		− 0.296	0.037	S
Number of hospitalizations		0.056	0.702	NS
Age of the child at illness onset		0.245	0.087	NS

*TEIQ* trait emotional intelligence, *NS* non-significant, *S* significant, *PANSS* positive and negative syndrome scale for schizophrenia

Table 3 shows the correlation between trait emotional intelligence in the offspring and clinical characteristics of schizophrenia of the parent; paternal age, age of the child at the onset of parent illness, duration of illness, and number of hospitalizations. There was significant negative correlation between duration of illness and TEIQ score

##### Relation between trait emotional intelligence of the offspring (TEIQue) global score and schizophrenia severity, type in the parent (total PANSS, subscales of PANSS)

No significant correlation found between schizophrenia severity (total PANSS score) and clinical symptoms (positive, negative, general psychopathology), with trait EI global score of the offspring. No relation between schizophrenia subtype and global TEIQue score (*f* 1.102, *p* value 0.358).

##### Relation between trait emotional intelligence subscales in the offspring (TEIQue subscale) and PANSS subscales in the parent

Low impulsivity in the offspring was negatively correlated to general psychopathology score of the parent. Parents with higher scores of pathology had children with higher impulsivity (*χ*^2^ − 0.326, *p* value 0.021).

##### Relation between trait emotional intelligence subscales in the offspring and schizophrenia subtypes of their parent

It was found that offspring of patients with paranoid schizophrenia had worse emotion regulation skills than offspring of parents with residual schizophrenia (***f*** 3.451, *p* value 0.024).

## Discussion

The main findings of this study that there is highly significant difference between offspring of a parent with schizophrenia and control regarding TEIQue subtests as emotion perception, self-esteem, low impulsivity, and emotion regulation. However, the global score of trait EI is not affected.

Those results are consistent with previous work by Lavoie, et al. (2013) as they reported impairment in emotion processing tasks including identification, perception, and regulation of emotion in relatives of schizophrenia [11]. Furthermore, another study by Allott (2015) prove that emotion recognition including emotion perception was impaired in first episode schizophrenia [12]. As participants had difficulty recognizing anger and disgust [12]. Also, by using ability EI test, Albacete, A. (2016) found that first-degree relatives presented with impairment across multiple emotion processing skills especially in their ability to accurately perceive, appraise, and express emotion. However, no study found specifically on Trait EI in relatives of schizophrenia [13].

The current study found that Trait EI in the offspring was related to duration of parental illness, while was not related to paternal age, onset, number of hospitalizations, which may be explained by (1) parent responsiveness and competence is affected by parent’s chronicity of illness, severity, and timing of parent’s mental illness with respect to the age of the child. (2) Longer duration of illness related to poor illness outcome, more environmental stressors, more societal complications, and burden. And this may be reflected on the offspring emotionality making them liable to more adverse life events. This finding was similar to previous study Kaasbøll (2021) that indicates that parental chronic illness is associated with an elevated risk for developing socio-emotional and behavioral problems in children, in particular internalizing symptoms [14].

While offspring Trait EI is not related to the number of hospitalizations of the parent, fewer hospitalizations was not associated with better Trait EI. This finding may be explained by the fact that hospitalizations indicate acute relapses which improves upon discharge, therefore no long contact with pathologizing symptoms of the parent.

Which is the opposite of what it is found before by Chen, J. (2021) as frequent hospitalizations predispose that child to more frequent family instability and possibility to be held in foster care or change in housing. Also, children became more liable for separation from the primary attachment figure, which may lead to affection of emotional wellbeing of the child and predisposes to high anxiety level and neuroticism in the children [15]. However, in Egypt that may be overcome by the role of healthy parent and family support by other members of the extended family usually encountered in Egypt.

While no correlation between schizophrenia severity (total PANSS score) and their children trait EI global TEIQue score. Also, none of the TEIQue global score or subscales were related to positive symptoms (*p* value 0.538) or negative symptoms (*p* value 0.503) scale.

That was opposite to what was expected. As some study prove that there is direct correlation between emotional intelligence of the parent and that of their children [16]; so, it was expected that severity of negative symptoms in the parent may affect emotional expression and involvement in the parent-child relationship, also parents prone to primary negative symptoms were known to have poorer social functioning with tendency to social withdrawal.

On the other hand, neuroanatomical predisposition to negative symptoms involves circuits of emotions which may be genetically transmitted to offspring’s brain and may be reflected in emotional development. However that could be modulated by environmental factors as warmth and support by other member of the extended family as in the current study.

While it was found that general psychopathology scale of PANSS of the parent is correlated with low impulsivity subscale of offspring, however not related to other subscales, while the domain of general psychopathology was usually linked to lack of judgement and insight [19].

Those findings may be explained by neuroanatomically dopamine deficiency in the prefrontal cortex. Impulsivity in schizophrenia was found to be associated with white matter tracts in the right dorsolateral prefrontal cortex and the right frontal pole may underlie dysfunctional impulse control [17].

In a study by Tikàsz et al. (2018), involved violent patients with schizophrenia showed that the DLPFC serves as a core region of an anger-related inhibitory cognitive control network [18]. Another study Jung et al. (2022) found that impulse-control difficulties experienced by schizophrenia patients may relate to deteriorated cognitive control function [17]. Therefore, affection of impulsivity in offspring is also related to emotion processing of anger.

Those findings were in light of previous research of high-risk offspring of bipolar disorder found to have higher impulsivity scores than healthy control subjects which suggest impulsivity as trait marker for vulnerability for both bipolar and mood disorders [21].

Why severity of schizophrenia in the parent did not affect trait emotional intelligence in the offspring, this may be explained by (1)

mild severity of the included sample as mean PANSS score was 37.82 ± 15.08. (2) The heritable impairment of emotions in schizophrenia is polygenic, which may need larger samples to be detected. (3) Children of parent with schizophrenia may be resilient or had another factors like environmental, sex, cognition, and temperament that may affect the probability of emotional impairments [22].

Also, there was limitations of the PANSS scale to reflect social functioning of the parent and may be not representative of clinical functioning [23]**,** while the use of self-perception emotional assessment tool in high-risk offspring may give inaccurate results in already verbally impaired children, one of the drawbacks of self-rating scale in children. Different research results of emotional intelligence impairments (impaired emotion processing tasks) in first-degree relative studies like previous meta-analysis by Lavoie et al. (2013) due to different sample age as they involve siblings, parents, and offspring, and they found that parents had poorer global EI scores than siblings and offspring which may affect total result [11].

Lastly, it was found that offspring of a parent with paranoid schizophrenia had poorer emotion regulation as a trait than offspring of a parent with residual schizophrenia. Rarely, there is study between symptomatology of schizophrenia in the parent and its effect on offspring development. However, Rutter (1984) mentioned that children may be involved in their parent’s paranoid themes [24]. While those results may be explained by what is known about parenting style in schizophrenia that it is characterized by low warmth and less expression of emotion while high in control [20]. Also, paranoid delusional themes may be associated with higher control. High-control parenting associated with more emotional dysregulation in the children.

### Strengths of this study

To our knowledge, this is one of few studies that study emotional intelligence in high-risk children and adolescent offspring of parent with schizophrenia and the first in Egypt. Also, it is one of the few studies which used trait emotional intelligence TEIQue scale in first degree relatives of schizophrenia.

### Limitations of this study

Limitations of this study include (1) small sample size due to difficult enrolment of subjects due to COVID-19 restrictions; (2) limitation of self-report TEIQue to asses all integrative models of EI constructs; so, limited social cognition accurate assessments and other tests measuring ability and integrative models of EI could have been done; (3) this may highlight the need for multiple integrative battery for assessing social cognition in both patients and their relatives, to differentiate both genetic and environmental factors; (4) presence of multiple variables in the parent must be included in further studies. Role of medications given to the parents, difference in the effect between mother or father illness, number of episodes of active illness, and relation between each symptom and its effect on offspring.

Further research is needed in the area of emotional intelligence in high-risk offspring, as it reflect trait marker of schizophrenia in their relatives. Also, for early intervention aiming to improve quality of life of families with schizophrenia, with involvement of offspring as stakeholder for the Quality Of life service of schizophrenic parents. Also, questions about the most effective intervention for high-risk children to reduce their morbidity.

## Conclusions

There is a possibility that children of parents with schizophrenia had impaired trait emotional intelligence in some of the facets, especially emotion perception and regulation. This may enhance a new trial of early intervention in those children that improve their outcome level of functioning.

### Recommendations

Children at risk need to be focus of attention of mental health services at Egypt.

## Data Availability

The datasets used and/or analyzed during the current study are available from the corresponding author on reasonable request.

## Abbreviations

EI: Emotional intelligence
IQ: Intelligent quotient
OPC: Outpatient clinic
TEIQue: Trait emotional intelligence questionnaire
DLPFC: Dorsolateral prefrontal cortex
COVID: Corona virus disease-19
PANSS: Positive and negative symptom scale
